# Comparative study between sorafenib and lenvatinib as the first‐line therapy in the sequential treatment of unresectable hepatocellular carcinoma in a real‐world setting

**DOI:** 10.1002/jgh3.12691

**Published:** 2021-12-17

**Authors:** Taito Fukushima, Manabu Morimoto, Makoto Ueno, Kousuke Kubota, Haruki Uojima, Hisashi Hidaka, Makoto Chuma, Kazushi Numata, Kota Tsuruya, Shunji Hirose, Tatehiro Kagawa, Nobuhiro Hattori, Tsunamasa Watanabe, Kotaro Matsunaga, Kouji Yamamoto, Katsuaki Tanaka, Shin Maeda

**Affiliations:** ^1^ Hepatobiliary and Pancreatic Oncology Kanagawa Cancer Center Yokohama Kanagawa Japan; ^2^ Department of Gastroenterology Yokohama City University Graduate School of Medicine Yokohama Kanagawa Japan; ^3^ Department of Gastroenterology, Internal Medicine Kitasato University School of Medicine Sagamihara Kanagawa Japan; ^4^ Gastroenterological Center Yokohama City University Medical Center Yokohama Kanagawa Japan; ^5^ Division of Gastroenterology and Hepatology, Department of Internal Medicine Tokai University School of Medicine Isehara Kanagawa Japan; ^6^ Division of Gastroenterology and Hepatology, Department of Internal Medicine St. Marianna University School of Medicine Kawasaki Kanagawa Japan; ^7^ Division of Gastroenterology and Hepatology Kawasaki Municipal Tama Hospital Kawasaki Kanagawa Japan; ^8^ Department of Biostatistics, School of Medicine Yokohama City University Yokohama Kanagawa Japan; ^9^ Gastroenterology Division Hadano Red Cross Hospital Hadano Kanagawa Japan

**Keywords:** hepatocellular carcinoma, lenvatinib, molecular‐targeted therapy, propensity score matching, sorafenib

## Abstract

**Aims:**

There is a paucity of comparative data on the use of sorafenib and lenvatinib for unresectable hepatocellular carcinoma. We assessed the real‐world treatment outcomes between using sorafenib and lenvatinib for unresectable hepatocellular carcinoma in the multiple molecular‐targeted therapy era.

**Methods and Results:**

We enrolled 386 patients treated with sorafenib or lenvatinib as the first‐line therapy for unresectable hepatocellular carcinoma at multiple centers. Propensity score matching was performed to adjust for differences in baseline and tumor characteristics between the two groups. Propensity score matching identified 110 patients in each treatment group. The median overall survival was similar between lenvatinib and sorafenib (14.8 and 13.0 months, respectively; *P* = 0.352). The median progression‐free survival was longer with lenvatinib than with sorafenib (7.6 and 3.9 months, respectively; *P* < 0.001). The overall response rate (*P* < 0.001) and disease control rate (*P* = 0.015), as defined by the modified Response Evaluation Criteria in Solid Tumors, were significantly better with lenvatinib than with sorafenib. The median overall survival was longer in patients who received subsequent treatment than in those who did not in the sorafenib group (23.1 and 5.7 months, respectively; *P* < 0.001), whereas the median overall survival with or without subsequent treatment did not differ significantly in the lenvatinib group (17.8 and 14.7 months, respectively; *P* = 0.439).

**Conclusion:**

Overall survival with sorafenib and lenvatinib was not significantly different. However, patients who received subsequent treatments had longer overall survival than those who received only first‐line treatment with sorafenib, whereas lenvatinib did not show this effect.

## Graphical abstract

This study compares the outcomes of sorafenib and lenvatinib for unresectable hepatocellular carcinoma using propensity score matching. Overall survival with sorafenib and lenvatinib was not significantly different. However, patients who received subsequent treatments had longer overall survival than those who received only first‐line treatment with sorafenib, whereas lenvatinib did not show this effect.

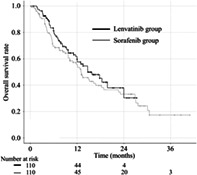

## Introduction

Hepatocellular carcinoma (HCC) is the fifth most common type of cancer worldwide and third most common cause of cancer‐related death.^1^ HCC is often diagnosed at intermediate or advanced stages, and one‐third of patients are diagnosed with advanced‐stage HCC.^2^, ^3^ Additionally, advanced‐stage HCC is known to have a poor prognosis and insufficiently effective therapies.

The multikinase inhibitors sorafenib and lenvatinib are the approved first‐line systemic treatments for unresectable HCC.^4^, ^5^, ^6^ Regorafenib, cabozantinib, and ramucirumab were approved in the second‐line setting.^7^, ^8^, ^9^ Moreover, a recent phase 3 study showed the benefits of a combination of atezolizumab and bevacizumab in terms of overall survival (OS) and progression‐free survival (PFS).^10^ Despite the availability of multiple drugs, which drugs should be used as primary treatment for advanced HCC and the optimal therapeutic sequence of drugs remain unclear.^11^, ^12^, ^13^ In the REFLECT study, the median OS was 13.6 months in the lenvatinib arm and 12.3 months in the sorafenib arm. However, these data were derived from a well‐selected patient population in a clinical trial; thus, real‐world evidence is required. Moreover, the clinical prognosis of sequential treatment with various combinations of treatment is an unresolved clinical question.

Here, we investigated the treatment outcomes of patients with HCC who received sorafenib or lenvatinib as first‐line therapy in a real‐world setting. To investigate the results only from the era wherein sequential treatment using molecular‐targeted agents became available, we only included data from 2017 onward, when regorafenib became available in Japan.

## Methods

### Patients

From January 2017 to March 2020, we treated 386 consecutive patients with unresectable HCC, with sorafenib or lenvatinib as the first‐line therapy, at the following five institutions in the Kanagawa Liver study group: Kanagawa Cancer Center, Kitasato University Hospital, Yokohama City University Medical Center, Tokai University Hospital, and St. Marianna University School of Medicine Hospital. Clinical information and follow‐up data for these consecutive patients were obtained from medical records and examined retrospectively. The data cut‐off date was set for 28 August 2020.

The study protocol was approved by the respective Institutional Review Boards and was conducted per the Declaration of Helsinki (as revised in Fortaleza, Brazil, October 2013). The Institutional Review Boards waived the need for obtaining written informed consent because of the retrospective study design. All patient data were anonymized after data collection.

### Diagnosis

Patients were diagnosed with HCC based on at least one typical HCC image or pathological findings. Clinical diagnoses of HCC were established according to the diagnostic criteria of the European Association for the Study of the Liver guidelines.^14^ These guidelines recommend computed tomography (CT) or magnetic resonance imaging (MRI) for diagnosis due to their high sensitivities. Pathological diagnosis should be performed based on the International Consensus recommendations using the required histological and immunohistological analyses.

### Treatment

The European Association for the Study of the Liver guidelines recommend both sorafenib and lenvatinib as first‐line chemotherapy for advanced HCC.^14^ Accordingly, the treatment decision is made at the discretion of the attending physicians. Patients received oral sorafenib 400 mg twice daily or oral lenvatinib 12 mg/day (for ≥60 kg body weight) or 8 mg/day (for <60 kg body weight). Modification of the starting dose was allowed, depending on the clinical situation, at the discretion of the attending physicians. Treatment was discontinued when unacceptable adverse events or significant clinical tumor progression was observed.

### Assessment of the treatment response

Response assessment using CT or MRI was performed every 6–8 weeks or whenever there was a sign or symptom suggestive of tumor progression. Radiological assessments were evaluated according to both the Response Evaluation Criteria in Solid Tumors (RECIST) 1.1 and the modified RECIST (mRECIST).^15^, ^16^

### Monitoring liver function

Liver function was evaluated by the Child–Pugh class score^17^ and albumin–bilirubin (ALBI) score, which was calculated based on serum albumin and total bilirubin values as follows: (ALBI score = (0.66 × log10 bilirubin [μmol/L]) + [−0.085 × albumin^1^]), defined by the following scores: ≤−2.60 = grade 1, >−2.60 to ≤−1.39 = grade 2, and > −1.39 = grade 3.^18^

### Statistical analyses

Continuous parameters between the groups were analyzed using the Mann–Whitney U test and categorical parameters using Fisher's exact or the *χ* ^2^ test. *P* values < 0.05 were considered statistically significant. OS and PFS were calculated using the Kaplan–Meier method, and differences were evaluated using Cox's proportional hazards regression model.

To reduce patient selection bias, we used propensity score matching (PSM) between the sorafenib and lenvatinib groups. Propensity scores were estimated using age, sex, etiology, Barcelona Clinic Liver Cancer (BCLC) stage, serum albumin level, total bilirubin level, and AFP level. After the propensity scores had been established, we performed 1:1 matching. Statistical analyses were performed with R version 4.0.3 (The R Foundation for Statistical Computing, Vienna, Austria).

## Results

### Clinical characteristics

**Figure 1:**
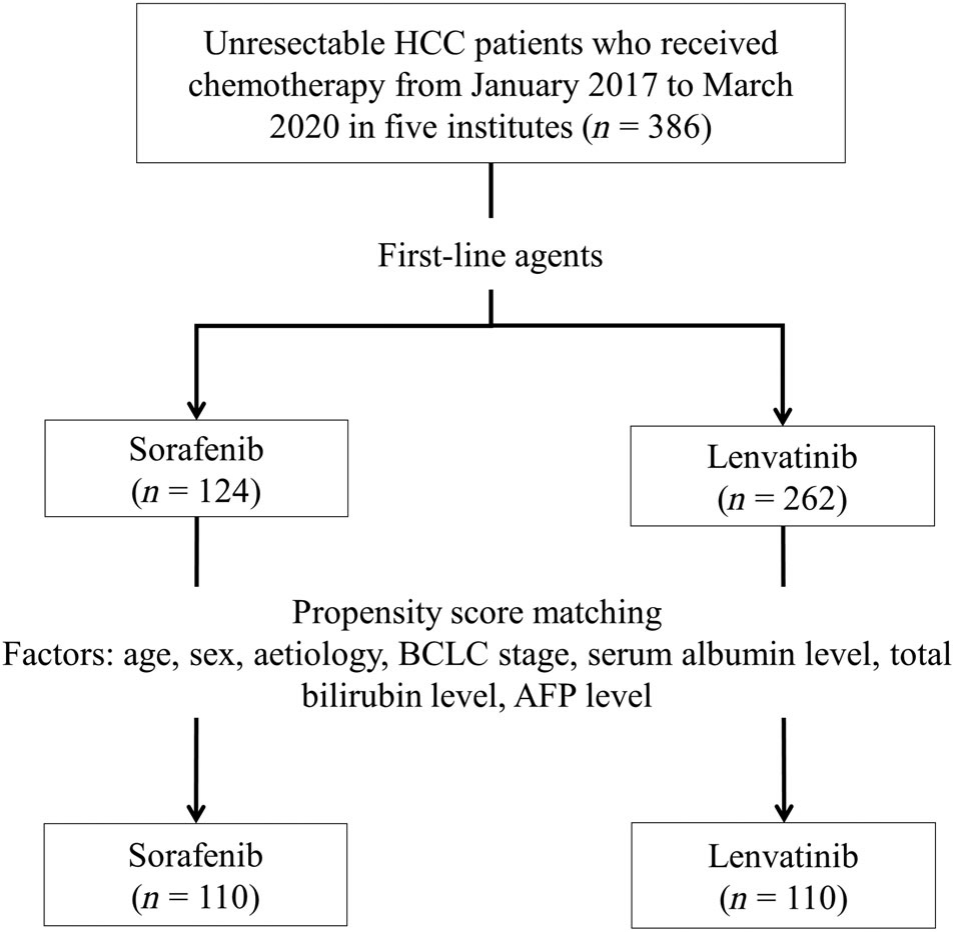
Flow diagram of this study. We evaluated 386 consecutive patients, who received chemotherapy, from five institutes. After propensity score matching (PSM), 110 patients in the sorafenib group and 110 patients in the lenvatinib group were selected, and the treatment efficacy and survival data were evaluated. AFP, alpha‐fetoprotein; BCLC, Barcelona Clinic Liver Cancer; HCC, hepatocellular carcinoma.

Before PSM, the study included 386 consecutive patients who received sorafenib or lenvatinib as first‐line treatment for HCC (Fig. 1). Baseline characteristics of the patients are shown in Table S1, Supporting information. There was a significant difference between the two groups concerning etiology (*P* < 0.001), extrahepatic metastasis (*P* = 0.040), and total bilirubin (*P* = 0.030). Characteristics of patients after PSM to account for imbalances in the baseline characteristics are shown in Table 1. PSM identified 110 patients from each treatment group and successfully matched the two treatment groups regarding etiology and total bilirubin. In contrast, extrahepatic metastasis was left unmatched.

**Table 1:** Baseline characteristics after propensity score matching (PSM)

	Sorafenib (*n* = 110)	Lenvatinib (*n* = 110)	*P*‐value
Age (years)^†^	72.0 (67.0–78.0)	73.0 (67.3–78.0)	0.474
Sex (male)^†^	94 (85.5%)	91 (82.7%)	0.580
Etiology			
HCV^†^	44 (40.0%)	36 (32.7%)	0.704
HBV^†^	27 (24.5%)	28 (25.5%)	
HCV and HBV^†^	1 (0.9%)	1 (0.9%)	
Other^†^	38 (34.5%)	45 (40.9%)	
BCLC stage			
A^†^	1 (0.9%)	2 (1.8%)	0.200
B^†^	47 (42.7%)	59 (53.6%)	
C^†^	62 (56.4%)	49 (44.5%)	
Macrovascular invasion^†^	28 (25.5%)	27 (24.5%)	0.876
Extrahepatic metastasis^†^	45 (40.9%)	27 (24.5%)	0.010
Child–Pugh class, B^†^	25 (22.7%)	24 (21.8%)	0.871
ALBI grade			
Grade 1^†^	40 (36.4%)	37 (33.6%)	0.353
Grade 2^†^	63 (57.3%)	70 (63.6%)	
Grade 3^†^	7 (6.4%)	3 (2.7%)	
AST (IU/L)^†^	40.0 (27.3–64.0)	43.0 (33.0–58.8)	0.273
Albumin (g/dL)^†^	3.7 (3.4–4.0)	3.7 (3.3–4.0)	0.541
Total bilirubin (mg/dL)^†^	0.7 (0.5–1.1)	0.8 (0.6–1.2)	0.324
Prothrombin time (%)^†^	88.0 (76.0–97.0)	86.5 (75.0–96.0)	0.947
AFP (ng/mL)^†^	67.4 (6.1–798.0)	63.7 (9.1–1144.0)	0.563

† Values are presented as *n*, *n* (%), or median (IQR [25th–75th percentile]).

AFP, alpha‐fetoprotein; ALBI, albumin–bilirubin; AST, aspartate aminotransferase; BCLC, Barcelona Clinic Liver Cancer; HBV, hepatitis B virus; HCV, hepatitis C virus; IQR, interquartile range.

### Survival analysis

**Figure 2:**
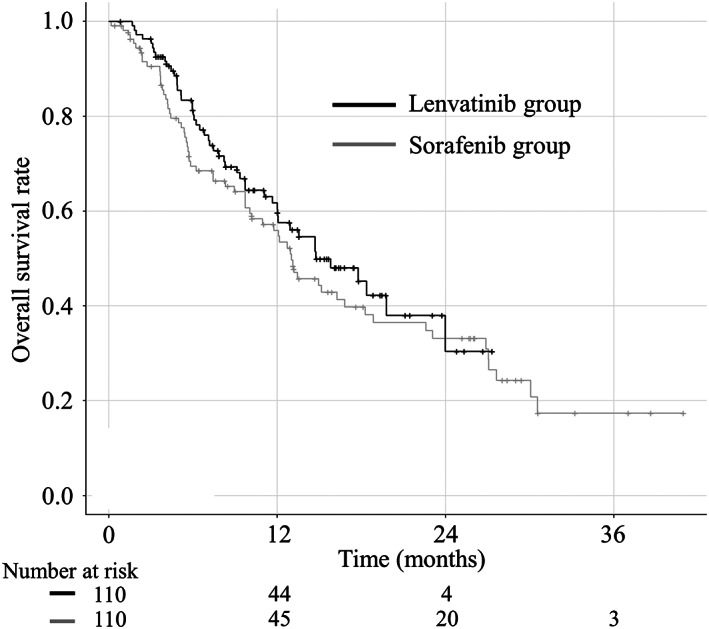
Kaplan–Meier analysis of overall survival (OS) in the sorafenib (gray line) and lenvatinib groups (black line) after propensity score matching (PSM). There are no significant differences between the groups (13.0 months in the sorafenib group and 14.8 months in the lenvatinib group; *P* = 0.352).

**Figure 3:**
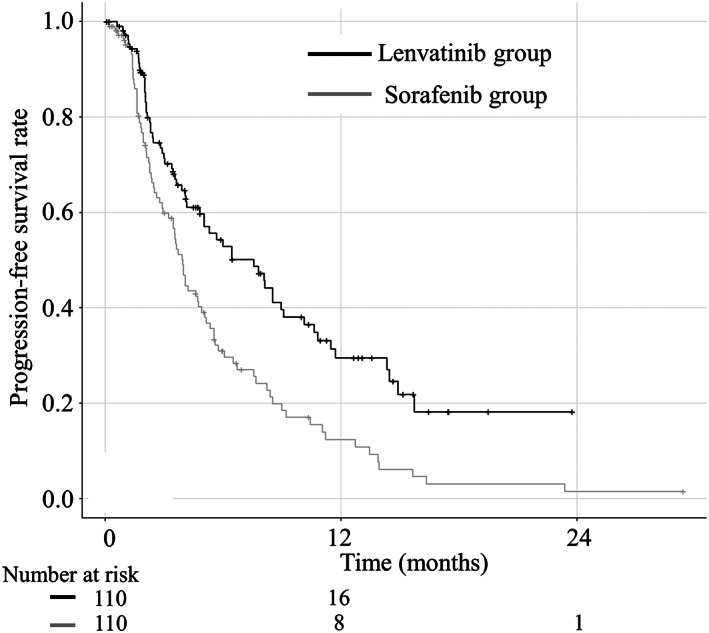
Kaplan–Meier analysis of progression‐free survival (PFS) in the sorafenib (gray line) and lenvatinib groups (black line) after the propensity score matching (PFS). The progression‐free survival (PFS) of the lenvatinib group is significantly longer than that of the sorafenib group (7.6 months *versus* 3.9 months, respectively; *P* < 0.001).

The median follow‐up period was 9.6 months. The median follow‐up period for the sorafenib and lenvatinib groups were 9.3 and 10.0 months, respectively (*P* = 0.789). The median OS did not differ significantly between the sorafenib and lenvatinib groups (Fig. 2; 13.0 and 14.8 months, respectively; hazard ratio [HR], 0.83; 95% confidence interval [CI], 0.57–1.22; *P* = 0.352). The median PFS for the lenvatinib group (7.6 months) was longer than that for the sorafenib group (3.9 months) (HR, 0.43; 95% CI, 0.27–0.69; *P* < 0.001; Fig. 3). Regarding Child–Pugh class A patients alone, the median OS did not significantly differ between groups (Fig. S1; 18.4 months in the lenvatinib group, 16.8 months in the sorafenib group; HR, 0.92; 95% CI, 0.58–1.48; *P* = 0.744).

### Radiological assessment

The results at the radiologic assessment according to the RECIST 1.1 and mRECIST are shown in Tables S2 and S3. In the sorafenib group, the overall response rate (ORR) was 6.4% and the disease control rate (DCR) was 48.2% according to the RECIST 1.1; the ORR was 7.3% and the DCR was 46.4% according to the mRECIST. In the lenvatinib group, the ORR was 25.5% and the DCR was 64.5% according to the RECIST 1.1; the corresponding values were 33.6% and 63.6%, respectively, according to the mRECIST. The ORR and DCR with lenvatinib were significantly better than with sorafenib, according to both the RECIST 1.1 (ORR; *P* < 0.001, DCR; *P* = 0.021) and mRECIST (ORR; *P* < 0.001, DCR; *P* = 0.015).

### Subsequent treatments

Subsequent treatments are shown in Table S4. Excluding patients who underwent first‐line treatment with sorafenib, 107 patients completed treatment, of whom 51 (47.7%) received subsequent treatment. The subsequent treatment was as follows: lenvatinib (n = 8; 15.7%), regorafenib (n = 32; 62.8%), ramucirumab (n = 2; 3.9%), transarterial chemoembolization (n = 3; 5.9%), hepatic arterial infusion (n = 4; 7.8%), and other treatments (n = 2; 3.9%). Excluding patients who underwent first‐line treatment with lenvatinib, 90 patients completed treatment, of whom 25 (27.8%) received subsequent treatment. The subsequent treatment is as follows: sorafenib (n = 11; 44.0%), regorafenib (n = 3; 12%), ramucirumab (n = 2; 8.0%), transarterial chemoembolization (n = 4; 16.0%), hepatic arterial infusion (n = 3; 12.0%), and other treatments (n = 2; 8.0%).

### Subsequent treatment survival analysis

**Figure 4:**
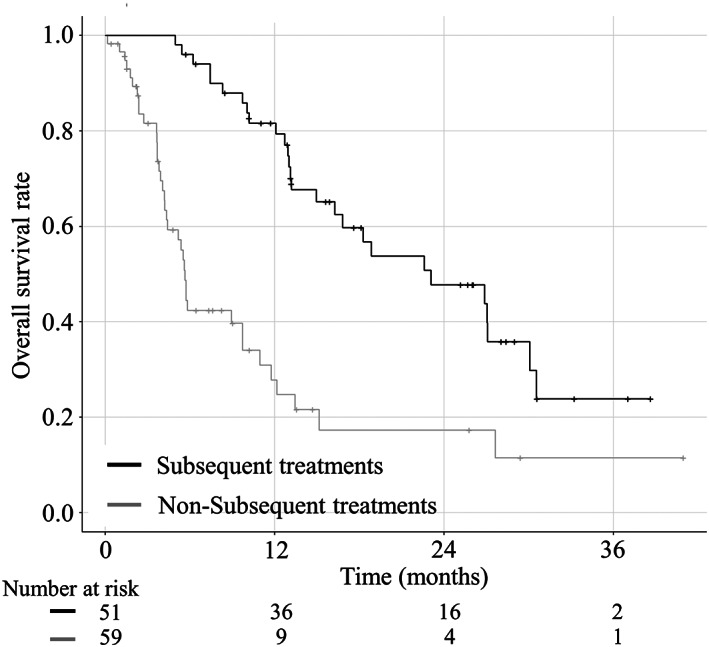
Kaplan–Meier analysis of overall survival (OS) of the sorafenib group according to subsequent (black line) and nonsubsequent treatments (gray line). The overall survival (OS) of the subsequent treatment group is significantly longer than that of the nonsubsequent treatment group (23.1 months *versus* 5.7 months, respectively; *P* < 0.001).

The median OS was longer in patients who received subsequent treatment (23.1 months) than in those in the sorafenib group (5.7 months) who did not receive subsequent treatment (HR, 0.29; 95% CI, 0.17–0.48; *P* < 0.001) (Fig. 4).

**Figure 5:**
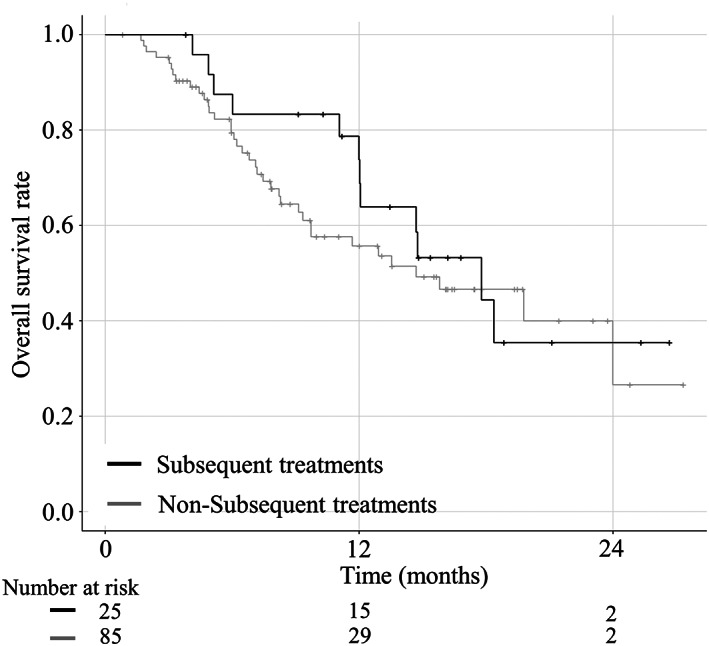
Kaplan–Meier analysis of overall survival (OS) in the lenvatinib group according to subsequent (black line) and nonsubsequent treatments (gray line). There are no significant differences between the treatments (17.8 months *versus* 14.7 months, respectively; *P* = 0.439).

The median OS did not differ significantly between the subsequent treatment (17.8 months) and nonsubsequent treatment (14.7 months) subgroups in the lenvatinib group (HR, 0.77; 95% CI, 0.40–1.48; *P* = 0.439) (Fig. 5). There were no significant differences in the baseline characteristics between the subsequent treatment and nonsubsequent treatment subgroups in the lenvatinib group (Table S5).

In Child–Pugh class A patients, the median OS was longer in patients who received subsequent treatment than in those in the sorafenib group who did not receive subsequent treatment (HR, 0.29; 95% CI, 0.17–0.48; *P* < 0.001) (Fig. S2). The median OS did not differ significantly between the subsequent treatment and nonsubsequent treatment subgroups in the lenvatinib group (HR, 0.77; 95% CI, 0.40–1.48; *P* = 0.439) (Fig. S3).

In the BCLC B and C stage patients, the median OS was longer in those who received subsequent treatment than in those in the sorafenib group who did not receive subsequent treatment (BCLC B: HR, 0.20; 95% CI, 0.07–0.52; *P* < 0.05, BCLC C: HR, 0.28; 95% CI, 0.15–0.53; *P* < 0.001). The median OS did not differ significantly between the subsequent treatment and nonsubsequent treatment subgroups in the lenvatinib group (BCLC B: HR, 0.52; 95% CI, 0.21–1.33; *P* = 0.172, BCLC C: HR, 1.07; 95% CI, 0.42–2.7; *P* = 0.888).

Regarding the comparison of survival limited to the subsequent treatment populations, the OS was significantly longer in Child–Pugh class A patients (HR, 2.93; 95% CI, 1.26–6.82; *P* < 0.05), but not significantly different in patients with Child–Pugh score 5 (HR, 1.32; 95% CI, 0.69–2.53; *P* = 0.395) and ALBI Grade score 1 (HR, 1.7; 95% CI, 0.89–3.22; *P* = 0.107).

### Radiological assessment of subsequent treatment

Radiological responses to subsequent treatment are shown in Tables S6 and S7. In the sorafenib group, the ORR was 7.8% and the DCR was 45.0% according to the RECIST 1.1; the ORR was 11.8% and the DCR was 47.1% according to the mRECIST. In the lenvatinib group, the ORR was 8.0% and the DCR was 40% according to the RECIST 1.1; the corresponding values were 8.0% and 36.0%, respectively, according to the mRECIST.

### Monitoring liver function

Changes in the Child–Pugh class scores during treatment are shown in Figures S4 and S5. The Child–Pugh class scores before treatment was class A in 85 patients (77.3%) and class B in 25 (22.7%) treated with sorafenib as a first‐line agent. The Child–Pugh class scores at the end of the primary treatment were class A in 64 patients (59.8%), class B in 33 (30.8%), and class C in 10 (9.3%) in the sorafenib group. The ALBI scores before and at the end of the primary treatment were −2.46 and −1.97, respectively, in the sorafenib group. The Child–Pugh class scores before treatment were class A in 86 patients (78.2%) and class B in 24 (21.8%) treated with lenvatinib as a first‐line agent. The Child–Pugh class scores at the end of first‐line treatment were class A in 38 patients (42.2%), class B in 45 (50.0%), and class C in seven (7.8%) in the lenvatinib group. The ALBI scores before and at the end of the primary treatment were −2.34 and −1.67, respectively, in the lenvatinib group. Both groups showed significant worsening of the Child–Pugh class scores by the end of first‐line treatment compared with before treatment (*P* < 0.001 in the sorafenib group; *P* < 0.001 in the lenvatinib group).

## Discussion

This study investigated the efficacy of the two first‐line HCC treatment agents, sorafenib and lenvatinib, and efficacies of subsequent treatments. The median OS of patients treated with sorafenib was 13.0 months, and that of patients treated with lenvatinib was 14.8 months. These results appeared to be lower than those obtained in the Japanese subset of the phase 3 REFLECT trial (median OS of 17.8 months with sorafenib and 17.6 months with lenvatinib).^19^ This may be due to the backgrounds of the study cohorts. In the REFLECT study, all subjects were Child–Pugh class A, whereas we included more than 20% of Child–Pugh class B cases. Several studies have shown that liver function is the most important prognostic factor in patients receiving tyrosine kinase inhibitors.^20^, ^21^, ^22^ Moreover, the baseline serum AFP levels in the REFLECT study were 49.8 and 57.1 ng/mL in the sorafenib and lenvatinib groups, respectively, whereas the baseline serum AFP levels in our study were higher (at 67.4 and 63.7 ng/mL, respectively). High AFP is a predictor of worse OS.^23^ Moreover, we included many patients who were not eligible for the REFLECT trial, such as those with major portal vein invasion and a tumor occupying >50% of the liver volume. Despite these differences, our study was designed to evaluate the treatment outcomes of real‐world patients.

Recent studies compared the outcomes of sorafenib and lenvatinib using PSM. Nakano *et al*. compared the clinical results of sorafenib and lenvatinib after PSM.^24^ The majority of patients in the study were treated before regorafenib was approved. In contrast, our study cohorts have begun enrollment in 2017 when regorafenib was approved in Japan; our study is up to date with multiple types of chemotherapy available. Additionally, another study from Japan compared the clinical results of sorafenib and lenvatinib PSM.^25^ However, that study included Child–Pugh class A cases alone. As our study aimed to reflect real‐world data, we included Child–Pugh B patients. Although Child–Pugh A is indicated for chemotherapy, the results of the analysis were similar for Child–Pugh A only in our cohort.

In this study, PFS was significantly longer, and the ORR and DCR were significantly higher in patients receiving lenvatinib than in those receiving sorafenib. These results were consistent with those of the REFLECT study.^6^, ^19^ Despite these benefits of lenvatinib use over sorafenib use, there was no significant difference in OS between the two cohorts in our population. There is a significant correlation between OS and postprogression survival (PPS). Kondo *et al*. reported that subsequent treatment after sorafenib contributed to a longer OS and PPS in patients with unresectable HCC.^26^ Another report indicated that the correlation between median OS and median time to progression was weak, while that between median OS and median PPS was strong.^27^ Our results suggested that patients who received lenvatinib did not show a better PPS due to inadequate subsequent treatment transition.

Additionally, our study analyzed the changes in the Child–Pugh class scores during treatment. Liver function deterioration at the end of first‐line treatment was more evident in patients using lenvatinib. However, since sorafenib and lenvatinib have different treatment durations, it is difficult to evaluate deterioration in liver function due to treatment by comparing the Child–Pugh class scores at the end of first‐line treatment. Hence, our result might not indicate that lenvatinib worsened liver function more than sorafenib during the treatment course, although it was possible that liver function deterioration in the lenvatinib group, which occurred during first‐line treatment, prevented patients from transitioning to subsequent treatment.

Another significant study finding was that patients who received subsequent treatments had a prolonged survival time compared with those who received first‐line treatment alone in the sorafenib group; however, this was not noted in the lenvatinib group. This may be due to the drug compatibility between the first and second‐line agents and differences in the rates of subsequent treatment between the sorafenib and lenvatinib groups. The evidence for second‐line treatment after sorafenib is well established, and three agents—regorafenib, ramucirumab, and cabozantinib—have shown clinical benefits in phase 3 trials.^7^, ^8^, ^9^ In contrast, unlike sorafenib, second‐line treatment after lenvatinib has not been firmly established—several studies have been reported^28^, ^29^, ^30^ but were based on a small number of patients, leaving insufficient evidence. In addition, there were a few options for subsequent treatment in the lenvatinib group because cabozantinib was not a treatment option during the study period. Considering these observations, we hypothesized that patients in the lenvatinib group might have fewer second‐line treatment options than those in the sorafenib group. Furthermore, it is difficult to determine whether a single agent or sequential treatment is superior before treatment in the lenvatinib group because there were no significant differences in the baseline characteristics. Similarly, we showed that among patients who received subsequent treatment, the OS was significantly longer in Child–Pugh class A patients. This result indicates that treatment in Child–Pugh class A patients should be tailored based on sequential therapy.

Based on the IMBrave150 trial, atezolizumab plus bevacizumab treatment is recommended as a first‐line therapy.^10^ Consequently, sorafenib and lenvatinib are positioned as second‐line therapies. Yoo *et al*. reported the clinical outcomes of subsequent treatment after progression on atezolizumab plus bevacizumab; their results were consistent with ours, although their results were based on second‐line treatment.^31^ Therefore, our results should be considered when choosing the most appropriate subsequent treatment for second‐line therapy. Considering the poor outcomes of patients who received first‐line treatment alone with sorafenib and the unfeasibility of proceeding to third‐line treatment for all cases, lenvatinib may be the best treatment option after atezolizumab plus bevacizumab treatment.

The study limitations include its retrospective nature, which does not allow for exclusion of unintended bias. Additionally, the median follow‐up period (9.6 months) was relatively short, as compared with that of the global phase 3 REFLECT trial (27.7 months).^6^ In particular, the observation period for lenvatinib is short because the drug was only approved in 2018 in Japan. Longer‐term observations may prolong the survival due to the introduction of subsequent treatment in many cases. The treatment options for advanced HCC changed during the observation period, which was another study limitation. Although some patients could not tolerate sorafenib or receive regorafenib, lenvatinib was not available in Japan until 2018. Thus, some patients in the sorafenib group could not receive subsequent treatment. This may have affected the OS of patients who received first‐line treatment alone with sorafenib. If possible, the study should have been limited to 2018 and beyond, when lenvatinib was approved, although the lack of data and the small number of patients render such analysis difficult. Therefore, prospective randomized studies with sufficient observational periods are needed to determine which treatment is optimal as first‐line treatment.

In conclusion, this retrospective multicenter study showed that the OS does not differ significantly between matched patients treated with sorafenib or lenvatinib as a first‐line treatment; however, lenvatinib showed statistically significant improvement in the PFS, ORR, and DCR. Moreover, this study showed that patients who received subsequent treatments after sorafenib had more prolonged survival than those who received first‐line treatment alone with sorafenib. Our results provide evidence for establishing sequential treatments of molecular‐targeted therapy in patients with advanced HCC.

### Ethics approval statement

The study protocol was approved by the Institutional Review Board of Kanagawa Cancer Center (2020–20) and the Institutional Review Boards of the respective institutions involved, and was conducted per the Declaration of Helsinki (as revised in Fortaleza, Brazil, October 2013).

### Patient consent statement

The relevant Institutional Review Boards waived the need for obtaining written informed consent because of the retrospective design. All patient data were anonymized after data collection.

## Supporting information


**Figure S1.** In Child‐Pugh class A patients, the median OS did not differ significantly between the sorafenib and lenvatinib groups (18.4 months in the lenvatinib group and 16.8 months in the sorafenib group; hazard ratio [HR], 0.92; 95% confidence interval [CI], 0.58–1.48; *P*=0.744).

**Figure S2.** In Child‐Pugh class A patients, the median OS was longer in patients who received subsequent treatment than in those in the sorafenib group who did not receive subsequent treatment (HR, 0.29; 95% CI, 0.17–0.48; *P*<0.001).

**Figure S3.** In Child‐Pugh class A patients, the median OS did not differ significantly between the subsequent treatment and non‐subsequent treatment subgroups in the lenvatinib group (HR, 0.77; 95% CI, 0.40–1.48; *P*=0.439).

**Figure S4.** The percentages of the Child‐Pugh class classes of patients at the start and end of sorafenib treatment. At the start of sorafenib, 85 patients (77.3%) are Child‐Pugh class A, and 25 (22.7%) are Child‐Pugh class B. At the end of sorafenib treatment, 64 patients (59.8%) are Child‐Pugh class A, 33 (30.8%) are Child‐Pugh class B, and 10 (9.3%) are Child‐Pugh class C.

**Figure S5.** The percentage of the Child‐Pugh class classes of patients at the start of lenvatinib and end of lenvatinib treatment. At the start of lenvatinib, 86 patients (78.2%) are Child‐Pugh class A and 24 (21.8%) are Child‐Pugh class B. At the end of lenvatinib treatment, 38 patients (42.2%) are Child‐Pugh class A, 45 (50.0%) are Child‐Pugh class B, and 7 (7.8%) are Child‐Pugh class C.

**Table S1.** Baseline characteristics.
**Table S2.** Radiological assessment using RECIST 1.1.
**Table S3.** Radiological assessment using mRECIST.
**Table S4.** Subsequent treatment.
**Table S5.** Baseline characteristics in the lenvatinib group.
**Table S6.** Radiological assessment of subsequent treatments using RECIST 1.1.
**Table S7.** Radiological assessment of subsequent treatments using mRECIST.

## Data Availability

The data that support the findings of this study are available from the corresponding author upon reasonable request.
