# Supplementary figures and images for: Comparative study between sorafenib and lenvatinib as the first‐line therapy in the sequential treatment of unresectable hepatocellular carcinoma in a real‐world setting
Source: JGH Open. 2021 Dec 17;6(1):29–35. doi: 10.1002/jgh3.12691 (PMC8762625; doi:10.1002/jgh3.12691)

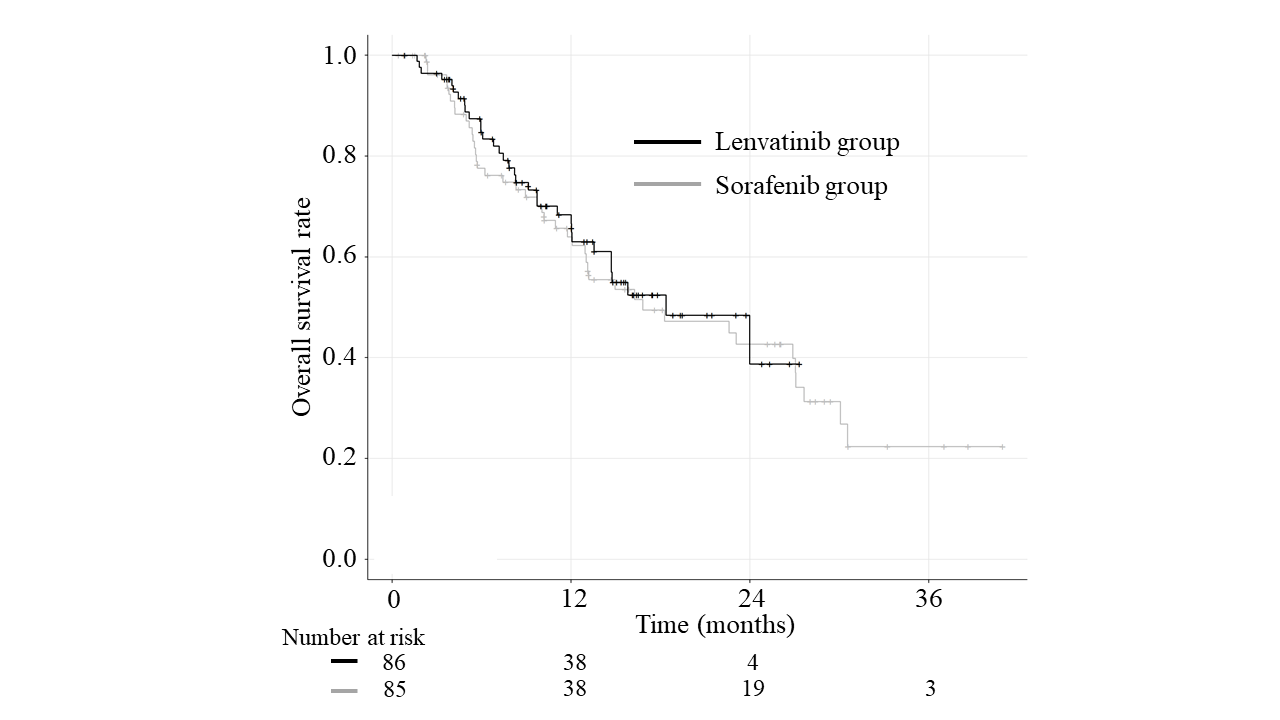

Supplement: Supplementary file 1 — Figure S1. In Child‐Pugh class A patients, the median OS did not differ significantly between the sorafenib and lenvatinib groups (18.4 months in the lenvatinib group and 16.8 months in the sorafenib group; hazard ratio [HR], 0.92; 95% confidence interval [CI], 0.58–1.48; P=0.744). [file JGH3-6-29-s002.tif]

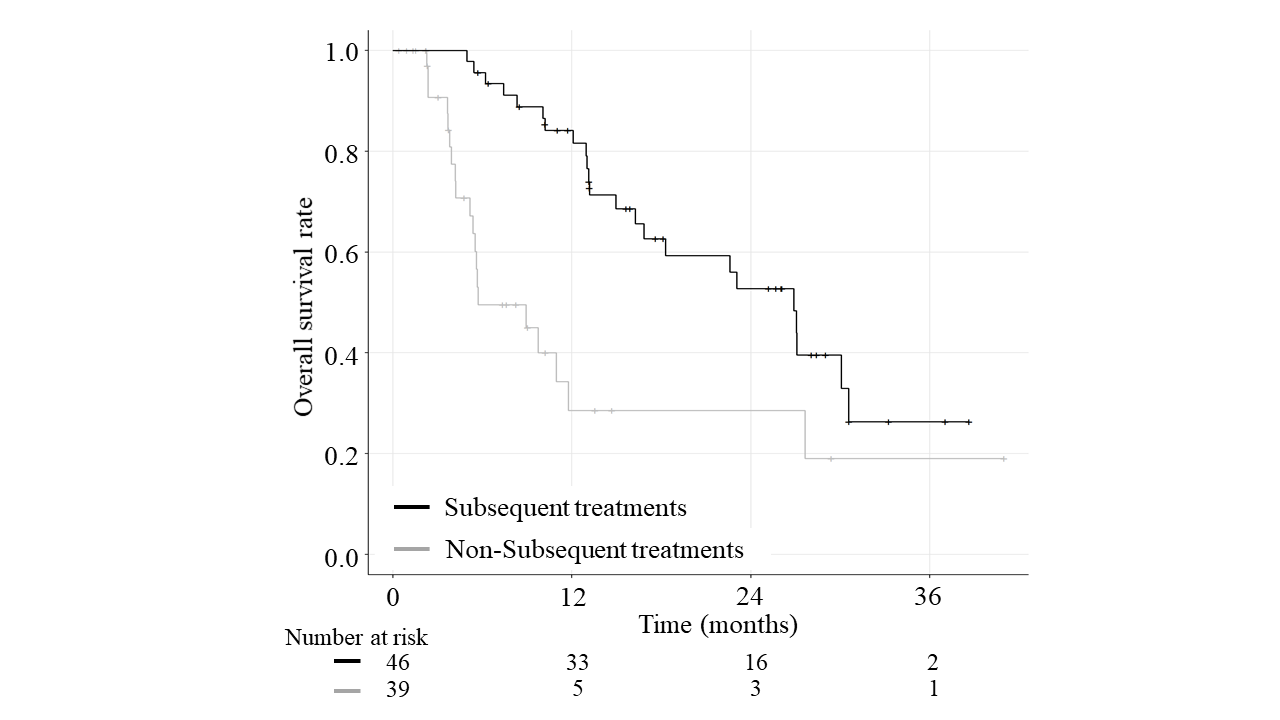

Supplement: Supplementary file 2 — Figure S2. In Child‐Pugh class A patients, the median OS was longer in patients who received subsequent treatment than in those in the sorafenib group who did not receive subsequent treatment (HR, 0.29; 95% CI, 0.17–0.48; P<0.001). [file JGH3-6-29-s003.tif]

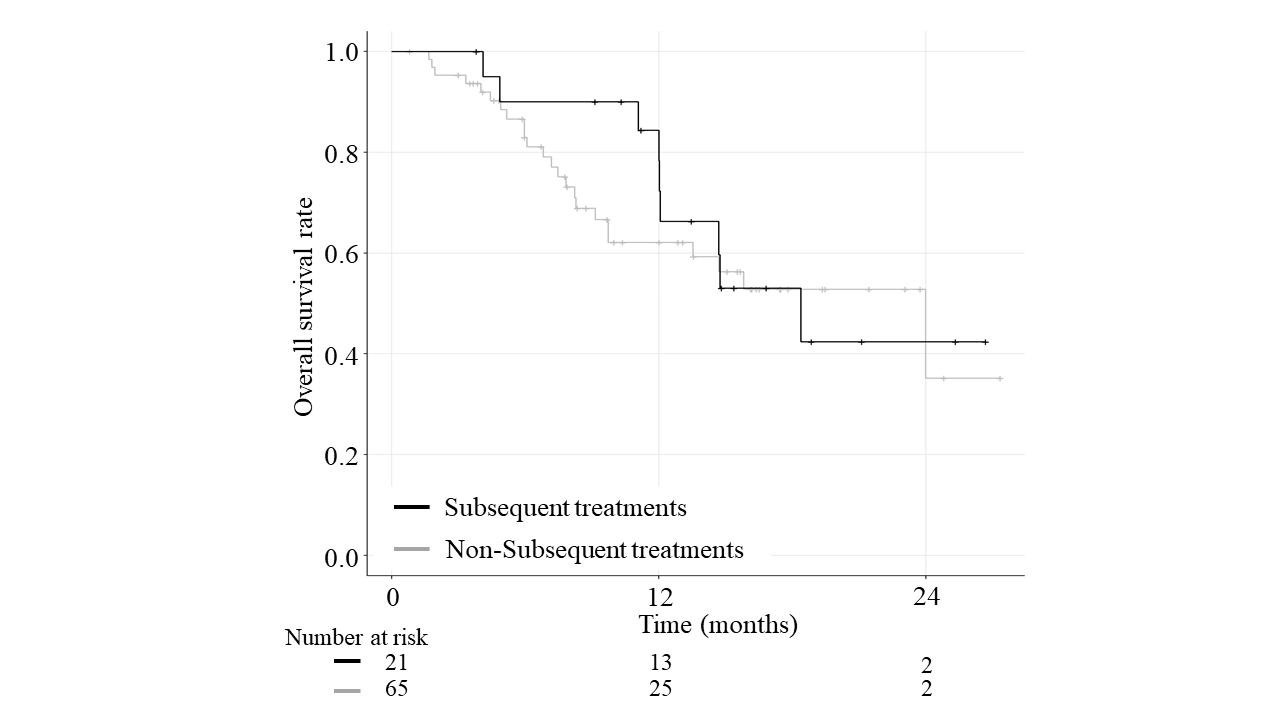

Supplement: Supplementary file 3 — Figure S3. In Child‐Pugh class A patients, the median OS did not differ significantly between the subsequent treatment and non‐subsequent treatment subgroups in the lenvatinib group (HR, 0.77; 95% CI, 0.40–1.48; P=0.439). [file JGH3-6-29-s005.tif]

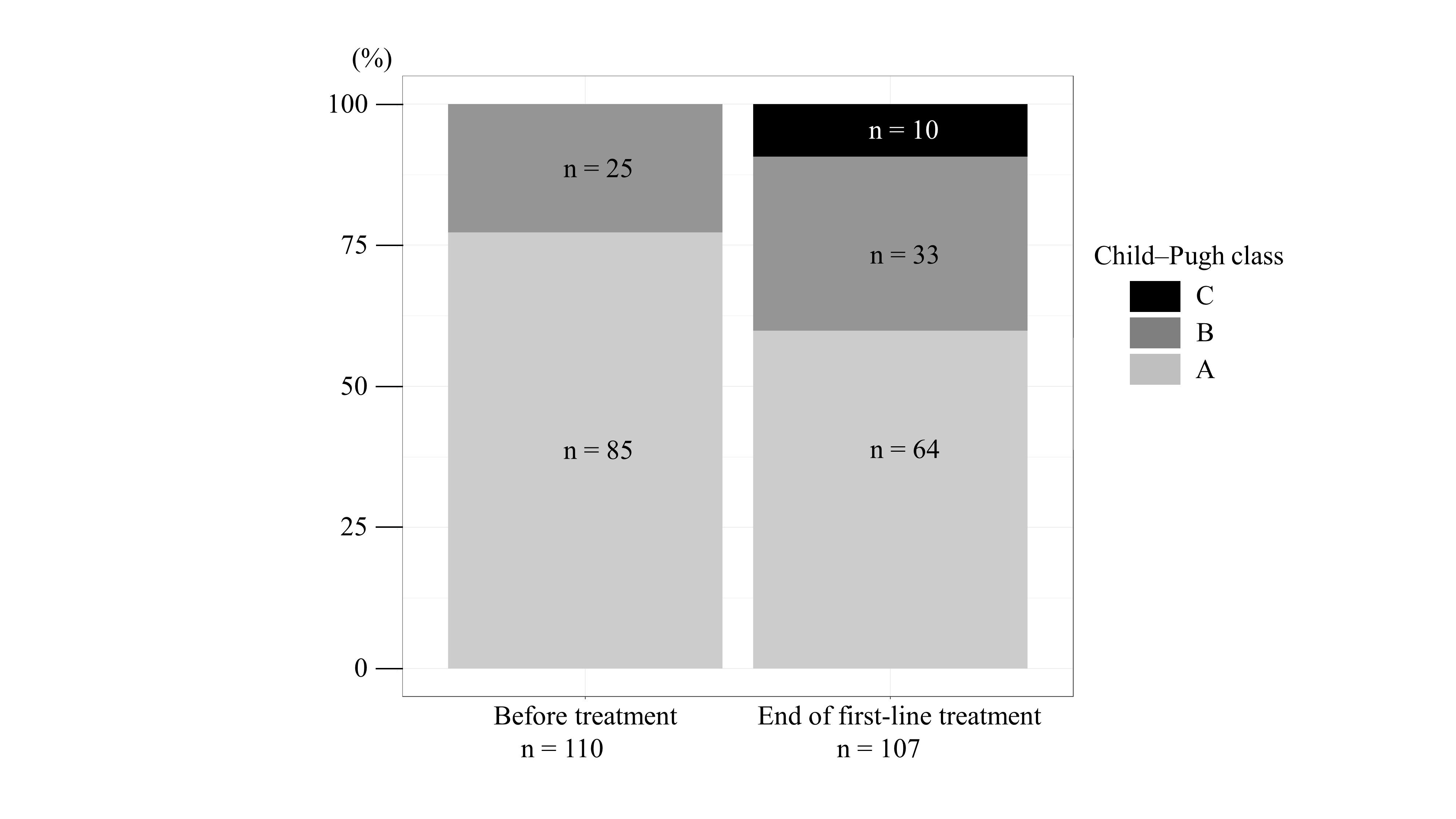

Supplement: Supplementary file 4 — Figure S4. The percentages of the Child‐Pugh class classes of patients at the start and end of sorafenib treatment. At the start of sorafenib, 85 patients (77.3%) are Child‐Pugh class A, and 25 (22.7%) are Child‐Pugh class B. At the end of sorafenib treatment, 64 patients (59.8%) are Child‐Pugh class A, 33 (30.8%) are Child‐Pugh class B, and 10 (9.3%) are Child‐Pugh class C. [file JGH3-6-29-s004.tif]

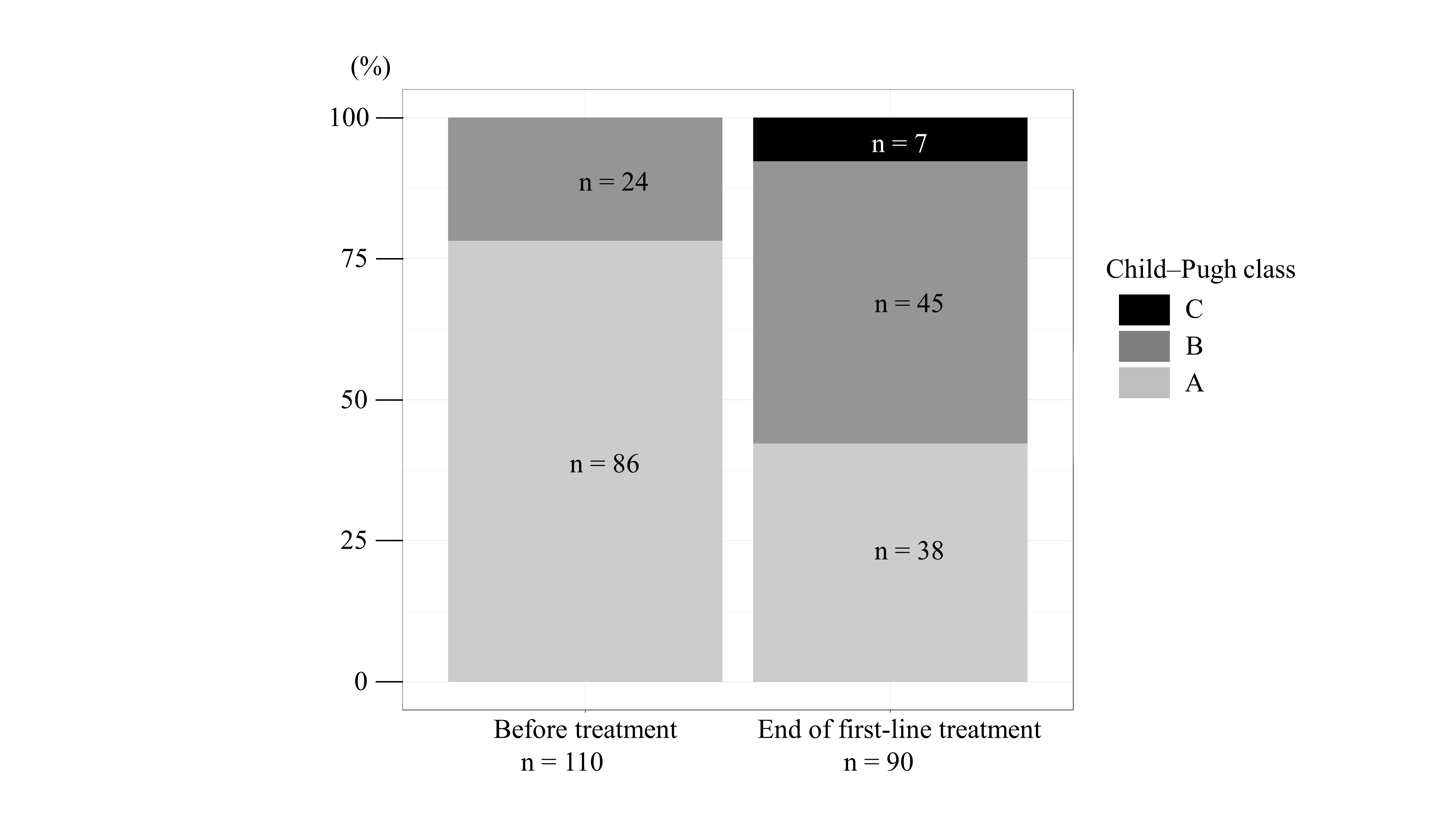

Supplement: Supplementary file 5 — Figure S5. The percentage of the Child‐Pugh class classes of patients at the start of lenvatinib and end of lenvatinib treatment. At the start of lenvatinib, 86 patients (78.2%) are Child‐Pugh class A and 24 (21.8%) are Child‐Pugh class B. At the end of lenvatinib treatment, 38 patients (42.2%) are Child‐Pugh class A, 45 (50.0%) are Child‐Pugh class B, and 7 (7.8%) are Child‐Pugh class C. [file JGH3-6-29-s001.tif]
