# Supplementary material for: Comparative study between sorafenib and lenvatinib as the first‐line therapy in the sequential treatment of unresectable hepatocellular carcinoma in a real‐world setting
Source: JGH Open. 2021 Dec 17;6(1):29–35. doi: 10.1002/jgh3.12691 (PMC8762625; doi:10.1002/jgh3.12691)
Supplement: Supplementary file 6 — Table S1. Baseline characteristics. Table S2. Radiological assessment using RECIST 1.1. Table S3. Radiological assessment using mRECIST. Table S4. Subsequent treatment. Table S5. Baseline characteristics in the lenvatinib group. Table S6. Radiological assessment of subsequent treatments using RECIST 1.1. Table S7. Radiological assessment of subsequent treatments using mRECIST. [file JGH3-6-29-s006.docx]

**Table S1.** Baseline characteristics

|  | Sorafenib  n=124 | Lenvatinib  n=262 | P-value |
| --- | --- | --- | --- |
| Age, years† | 72.5 (67.0–78.0) | 73.0 (66.0–79.0) | 0.581 |
| Sex, male† | 105 (84.7%) | 215 (82.1%) | 0.524 |
| Etiology  HCV†  HBV†  HCV and HBV†  Other† | 45 (36.6%)  29 (23.4%)  10 (8.1%)  40 (32.3%) | 91 (34.7%)  47 (19.9%)  1 (0.4%)  123 (46.9%) | <0.001 |
| BCLC stage  A†  B†  C† | 1 (0.8%)  55 (44.4%)  68 (54.8%) | 3 (1.1%)  139 (53.1%)  120 (45.8%) | 0.250 |
| Macrovascular invasion† | 30 (24.2%) | 59 (22.5%) | 0.715 |
| Extrahepatic metastasis† | 50 (40.3%) | 78 (29.8%) | 0.040 |
| Child‒Pugh class, B† | 25 (20.2%) | 52 (19.8%) | 0.943 |
| ALBI grade  Grade 1†  Grade 2†  Grade 3† | 48 (38.7%)  69 (55.6%)  7 (5.6%) | 99 (37.8%)  158 (60.3%)  5 (1.9%) | 0.128 |
| AST, IU/L† | 41.0 (28.0–63.3) | 39.0 (31.00–60.0) | 0.945 |
| Albumin, g/dL† | 3.7 (3.4–4.1) | 3.7 (3.3–4.1) | 0.895 |
| Total bilirubin, mg/dL† | 0.7 (0.5–1.0) | 0.8 (0.6–1.2) | 0.030 |
| Prothrombin time, %† | 89.0 (77.5–97.0) | 88.0 (75.0–99.0) | 0.787 |
| AFP, ng/mL† | 72.3 (6.6–854.1) | 51.1 (6.1–1310.0) | 0.979 |

†Values are presented as n, n (%), or median (IQR [25^th^–75^th^ percentile]).

Abbreviations: AFP, alpha-fetoprotein; ALBI, albumin‒bilirubin; AST, aspartate aminotransferase; BCLC, Barcelona Clinic Liver Cancer; HBV, hepatitis B virus; HCV, hepatitis C virus; IQR, interquartile range.

**Table S2.** Radiological assessment using RECIST 1.1

|  | Sorafenib  n=110 | Lenvatinib  n=110 | P-value |
| --- | --- | --- | --- |
| CR† | 1 (0.9%) | 4 (3.6%) |  |
| PR† | 6 (5.5%) | 24 (21.8%) |  |
| SD† | 46 (41.8%) | 43 (39.1%) |  |
| PD† | 37 (33.6%) | 28 (25.5%) |  |
| NE† | 20 (18.2%) | 11 (10.0%) |  |
| ORR (CR+PR) † | 7 (6.4%) | 28 (25.5%) | <0.001 |
| DCR (CR+PR+SD) † | 53 (48.2%) | 71 (64.5%) | 0.021 |

†Values are presented as n and n (%).

Abbreviations: CR, complete response; DCR, disease control rate; NE, not evaluated; ORR, overall response rate; PD, progressive disease; PR, partial response; SD, stable disease; RECIST, Response Evaluation Criteria in Solid Tumors.

**Table S3.** Radiological assessment using mRECIST

|  | Sorafenib  n=110 | Lenvatinib  n=110 | P-value |
| --- | --- | --- | --- |
| CR† | 1 (0.9%) | 5 (4.5%) |  |
| PR† | 7 (6.4%) | 32 (29.1%) |  |
| SD† | 43 (39.1%) | 33 (30.0%) |  |
| PD† | 34 (30.9%) | 26 (23.6%) |  |
| NE† | 25 (22.7%) | 14 (12.7%) |  |
| ORR (CR+PR) † | 8 (7.3%) | 37 (33.6%) | <0.001 |
| DCR (CR+PR+SD) † | 51 (46.4%) | 70 (63.6%) | 0.015 |

†Values are presented as n and n (%).

Abbreviations: CR, complete response; DCR, disease control rate; NE, not evaluated; ORR, overall response rate; PD, progressive disease; PR, partial response; SD, stable disease; mRECIST, modified Response Evaluation Criteria in Solid Tumors.

**Table S4.** Subsequent treatment

| Subsequent treatments | First-line treatment | |
| --- | --- | --- |
|  | Sorafenib  n=51 | Lenvatinib  n=25 |
| Sorafenib† |  | 11 (44.0) |
| Lenvatinib† | 8 (15.7) |  |
| Regorafenib† | 32 (62.8) | 3 (12.0) |
| Ramucirumab† | 2 (3.9) | 2 (8.0) |
| Transarterial chemoembolization† | 3 (5.9) | 4 (16.0) |
| Hepatic arterial infusion† | 4 (7.8) | 3 (12.0) |
| Other treatments† | 2 (3.9) | 2 (8.0) |

†Values are presented as n and n (%).

**Table S5.** Baseline characteristics in the lenvatinib group

|  | Non-Subsequent treatment  n=85 | Subsequent treatment  n=25 | P-value |
| --- | --- | --- | --- |
| Age, years† | 72.0 (67.0–78.0) | 75.0 (71.0–79.0) | 0.327 |
| Sex, male† | 71 (83.5%) | 20 (80.0%) | 0.682 |
| Etiology  HCV†  HBV†  HCV and HBV†  Other† | 26 (30.6%)  24 (28.2%)  1 (1.2%)  34 (40.0%) | 10 (40.0%)  4 (16.0%)  0 (0.0%)  11 (44.0%) | 0.566 |
| BCLC stage  A†  B†  C† | 2 (2.4%)  44 (51.8%)  39 (45.9%) | 0 (0.0%)  15 (60.0%)  10 (40.0%) | 0.612 |
| Macrovascular invasion† | 20 (23.5%) | 7 (28.0%) | 0.648 |
| Extrahepatic metastasis† | 22 (25.9%) | 5 (20.0%) | 0.548 |
| Child‒Pugh class, B† | 20 (23.5%) | 4 (16.0%) | 0.423 |
| ALBI grade  Grade 1†  Grade 2†  Grade 3† | 28 (32.9%)  54 (63.5%)  3 ( 3.5%) | 9 (36.0%)  16 (64.0%)  0 ( 0.0%) | 0.626 |
| AST, IU/L† | 45.0 (33.0, 62.0) | 36.00 (33.0, 55.0) | 0.130 |
| Albumin, g/dL† | 3.70 (3.3, 4.0) | 3.60 (3.4, 4.1) | 0.683 |
| Total bilirubin, mg/dL† | 0.80 (0.6, 1.2) | 0.80 (0.6, 1.3) | 0.636 |
| Prothrombin time, %† | 85.0 (75.0, 95.0) | 92.0 (85.0, 101.0) | 0.073 |
| AFP, ng/mL† | 57.7 (10.0, 1426.0) | 76.6 (7.4, 507.9) | 0.478 |

†Values are presented as n, n (%), or median (IQR [25^th^–75^th^ percentile]).

Abbreviations: AFP, alpha-fetoprotein; ALBI, albumin‒bilirubin; AST, aspartate aminotransferase; BCLC, Barcelona Clinic Liver Cancer; HBV, hepatitis B virus; HCV, hepatitis C virus; IQR, interquartile range.

**Table S6.** Radiological assessment of subsequent treatments using RECIST 1.1

|  | Sorafenib  n=51 | Lenvatinib  n=25 |
| --- | --- | --- |
| CR† | 0 (0%) | 0 (0%) |
| PR† | 4 (7.8%) | 2 (8.0%) |
| SD† | 19 (37.3%) | 8 (32.0%) |
| PD† | 16 (31.4%) | 11 (44.0%) |
| NE† | 12 (18.2%) | 4 (16.0%) |
| ORR (CR+PR) † | 4 (7.8%) | 2 (8.0%) |
| DCR (CR+PR+SD) † | 23 (45.1%) | 10 (40.0%) |

†Values are presented as n and n (%).

Abbreviations: CR, complete response; DCR, disease control rate; NE, not evaluated; ORR, overall response rate; PD, progressive disease; PR, partial response; SD, stable disease; RECIST, Response Evaluation Criteria in Solid Tumors.

**Table S7.** Radiological assessment of subsequent treatments using mRECIST

|  | Sorafenib  n=51 | Lenvatinib  n=25 |
| --- | --- | --- |
| CR† | 0 (0%) | 0 (0%) |
| PR† | 6 (11.8%) | 2 (8.0%) |
| SD† | 18 (35.3%) | 7 (28.0%) |
| PD† | 14 (27.5%) | 11 (44.0%) |
| NE† | 13 (25.5%) | 5 (20.0%) |
| ORR (CR+PR) † | 6 (11.8%) | 2 (8.0%) |
| DCR (CR+PR+SD) † | 24 (47.1%) | 9 (36.0%) |

†Values are presented as n and n (%).

Abbreviations: CR, complete response; DCR, disease control rate; NE, not evaluated; ORR, overall response rate; PD, progressive disease; PR, partial response; SD, stable disease; mRECIST, modified Response Evaluation Criteria in Solid Tumors.
